# Empagliflozin normalizes the size and number of mitochondria and prevents reduction in mitochondrial size after myocardial infarction in diabetic hearts

**DOI:** 10.14814/phy2.13741

**Published:** 2018-06-21

**Authors:** Masashi Mizuno, Atsushi Kuno, Toshiyuki Yano, Takayuki Miki, Hiroto Oshima, Tatsuya Sato, Kei Nakata, Yukishige Kimura, Masaya Tanno, Tetsuji Miura

**Affiliations:** ^1^ Department of Cardiovascular, Renal and Metabolic Medicine Sapporo Medical University School of Medicine Sapporo Japan; ^2^ Department of Pharmacology Sapporo Medical University School of Medicine Sapporo Japan; ^3^ Department of Cellular Physiology and Signal Transduction Sapporo Medical University School of Medicine Sapporo Japan

**Keywords:** Acute myocardial infarction, diabetes mellitus, empagliflozin, mitochondria, SGLT2 inhibitor

## Abstract

To explore mechanisms by which SGLT2 inhibitors protect diabetic hearts from heart failure, we examined the effect of empagliflozin (Empa) on the ultrastructure of cardiomyocytes in the noninfarcted region of the diabetic heart after myocardial infarction (MI). OLETF, a rat model of type 2 diabetes, and its nondiabetic control, LETO, received a sham operation or left coronary artery ligation 12 h before tissue sampling. Tissues were sampled from the posterior ventricle (i.e., the remote noninfarcted region in rats with MI). The number of mitochondria was larger and small mitochondria were more prevalent in OLETF than in LETO. Fis1 expression level was higher in OLETF than in LETO, while phospho‐Ser637‐Drp1, total Drp1, Mfn1/2, and OPA1 levels were comparable. MI further reduced the size of mitochondria with increased Drp1‐Ser616 phosphorylation in OLETF. The number of autophagic vacuoles was unchanged after MI in LETO but was decreased in OLETF. Lipid droplets in cardiomyocytes and tissue triglycerides were increased in OLETF. Empa administration (10 mg/kg per day) reduced blood glucose and triglycerides and paradoxically increased lipid droplets in cardiomyocytes in OLETF. Empa suppressed Fis1 upregulation, increased Bnip3 expression, and prevented reduction in both mitochondrial size and autophagic vacuole number after MI in OLETF. Together with the results of our parallel study showing upregulation of SOD2 and catalase by Empa, the results indicate that Empa normalizes the size and number of mitochondria in diabetic hearts and that diabetes‐induced excessive reduction in mitochondrial size after MI was prevented by Empa via suppression of ROS and restoration of autophagy.

## Introduction

Recent clinical studies have shown that sodium glucose cotransporter 2 (SGLT2) inhibitors prevent cardiovascular events, particularly heart failure, in patients with type 2 diabetes mellitus (DM) (Zinman et al. 2015; Neal et al. 2017). The mechanism underlying the beneficial effects of SGLT2 inhibitors is currently under intensive investigation. In our previous studies (Takada et al. 2012; Murase et al. 2015), Otsuka Long‐Evans Tokushima Fatty rats (OLETF), a model of obese type 2 DM, mimicked patients with type 2 DM in terms of a significant increase in the mortality after acute myocardial infarction (MI) (Marso et al. 2007; De Luca et al. 2013). The mortality rate during a period of 48 h after acute MI was markedly higher in OLETF than in Long‐Evans‐Tokushima Otsuka rats (LETO), nondiabetic controls (Takada et al. 2012; Murase et al. 2015), despite preserved contractility and only mildly impaired relaxation of the left ventricle in OLETF (Takada et al. 2012; Kouzu et al. 2015). In our study conducted in parallel with the present study, treatment with empagliflozin for 2 weeks significantly reduced mortality after acute MI (Oshima et al. 2017), further supporting the cardioprotective effect of an SGLT2 inhibitor.

Various possible mechanisms by which an SGLT2 inhibitor reduces the incidence of heart failure and other cardiac events in diabetic patients have been proposed. The proposed mechanisms include improved cardiac energy metabolism by increased utilization of circulating ketone bodies (Mudaliar et al. 2016), attenuation of oxidative stress in the myocardium (de Leeuw and de Boer 2016) and vessels (Oelze et al. 2014), reduction in ventricular afterload by blood pressure lowering (Cherney et al. 2014), and inhibition of Na^+^–H^+^ exchange in cardiomyocytes (Bertero et al. 2017). In mouse models of diabetes, SGLT2 inhibitors improved ventricular diastolic function and attenuated ventricular hypertrophy (Habibi et al. 2017; Hammoudi et al. 2017; Joubert et al. 2017). Recently, we have focused on alterations caused by diabetes in a remote noninfarcted region of the heart after MI because the noninfarcted myocardium plays a critical role in acute adaptation to maintain cardiac output. We found that activation of autophagy, a stress‐responsive intracellular digestion system, in the noninfarcted region is blunted by diabetes, possibly contributing to insufficient mechanical compensation (Murase et al. 2015). In addition, we found that empagliflozin significantly improved ATP generation together with increased ketone utilization and upregulated superoxide dismutase 2 (SOD2) and catalase expression in the noninfarcted region of the heart after MI in OLETF (Oshima et al. 2017). On the other hand, effects of treatment with an SGLT2 inhibitor on ultrastructural changes in cardiomyocytes have not been examined in detail. Thus, the present study was designed to determine how diabetes and empagliflozin modify the ultrastructure of cardiomyocytes, mainly focusing on mitochondria, in the noninfarcted region of the heart with MI.

## Methods

### Animals

This study was conducted in strict accordance with the Guide for the Care and Use of Laboratory Animals published by National Research Council of the National Academies, USA (2011) and was approved by the Animal Use Committee of Sapporo Medical University. Male LETO and OLETF were purchased from Sankyo Labo Service Corporation (Tokyo, Japan). LETO and OLETF at ages of 25–30 weeks were used in all experiments as in our previous studies (Takada et al. 2012; Kouzu et al. 2015; Murase et al. 2015). Vehicle‐treated LETO and OLETF and empagliflozin‐treated OLETF were divided into two subgroups depending on whether a sham operation (Sham) or coronary ligation to induce MI was performed. Therefore, we compared six groups of animals: LETO‐Sham, LETO‐MI, OLETF‐Sham, OLETF‐MI, OLETF‐empagliflozin‐Sham (OLETF‐Empa‐Sham), and OLETF‐empagliflozin‐MI (OLETF‐Empa‐MI).

We selected a permanent coronary occlusion protocol, not an ischemia/reperfusion protocol, to prepare the same sizes of MI in all treatment groups. Our previous studies have shown that the size of MI after ischemia/reperfusion is significantly larger in OLETF than that in LETO (Miki et al. 2009; Hotta et al. 2010). Tanajak et al. (2018) recently reported that dapagliflozin limited MI size after ischemia/reperfusion in a rat model of insulin resistance. Thus, the use of an ischemia/reperfusion protocol in LETO and OLETF makes it difficult to critically assess the impact of diabetes or empagliflozin on cardiomyocytes in the noninfarcted region because of the difference in the loading condition on that region due to different MI sizes. Such intergroup differences in MI size may also result in differences in the survival rate after MI, leading to selection bias in tissue sampling. Permanent coronary occlusion consistently induces severe dyskinesis of the territory of the occluded artery regardless of the treatment in rats because of the very low coronary collateral flow level in this species (Maxwell et al. 1987; Hearse et al. 1988; Takada et al. 2012), and we have previously confirmed that MI sizes after permanent coronary artery occlusion were comparable in LETO and OLETF 48 h after MI (Murase et al. 2015).

### Administration of empagliflozin

Rats were pretreated with a vehicle (dimethyl sulfoxide and polyethylene glycol; 1:1 vol: vol) or empagliflozin (10 mg/kg per day) for 2 weeks. Empagliflozin was kindly provided by Boehringer Ingelheim (Ingelheim, Germany). The agents were administered to rats by use of osmotic minipumps (Alzet, Cupertino, CA).

### Induction of MI

Rats were fasted for 12 h and blood pressure and heart rate were measured in a conscious state by using a tail‐cuff system (BP‐98A, Softron, Tokyo, Japan) before surgery. The rats were prepared for induction of MI as in our previous studies (Takada et al. 2012; Murase et al. 2015). In brief, rats were placed in an induction chamber, and anesthesia was induced with isoflurane (5%) with careful monitoring of the level of anesthesia. After intubation, rats were ventilated with a rodent respirator (model 683, Harvard Apparatus, South Natick, MA) with supplementation of oxygen (1 L/min) and anesthesia by isoflurane (3–3.5%). After left thoracotomy, a marginal branch of the left coronary artery was permanently ligated by using a 5‐0 silk thread to induce MI. Sham‐operated rats served as controls. The surgical wounds were repaired, and the rats were returned to their cages.

### Sampling of myocardial tissues and blood

Since our previous studies showed that mortality during a period of 24–48 h after MI was significantly higher in OLETF than in LETO (Takada et al. 2012; Murase et al. 2015), 12 h after MI or the sham operation was selected as the time point for sampling of myocardial tissues and blood. Rats were kept fasted after surgery, and anesthesia and ventilation were performed as they were at the time of the first surgery. Blood pressure and heart rate were monitored by a catheter placed in the carotid artery, and blood was sampled via the arterial catheter. Blood glucose level was determined by using a Glutest‐mint (Sanwa Kagaku Kenkyusho, Nagoya, Japan). The chest was re‐opened and the hearts were excised and immediately immersed in ice‐cold saline. The myocardium in the noninfarcted region (posterior wall of the ventricle) was quickly excised in the saline, and half of the sample was fixed for electron microscopic analyses. The other half was frozen in liquid nitrogen and stored at −80°C until use for biochemical and gene expression analyses.

### Electron microscopy

**Figure 1:**
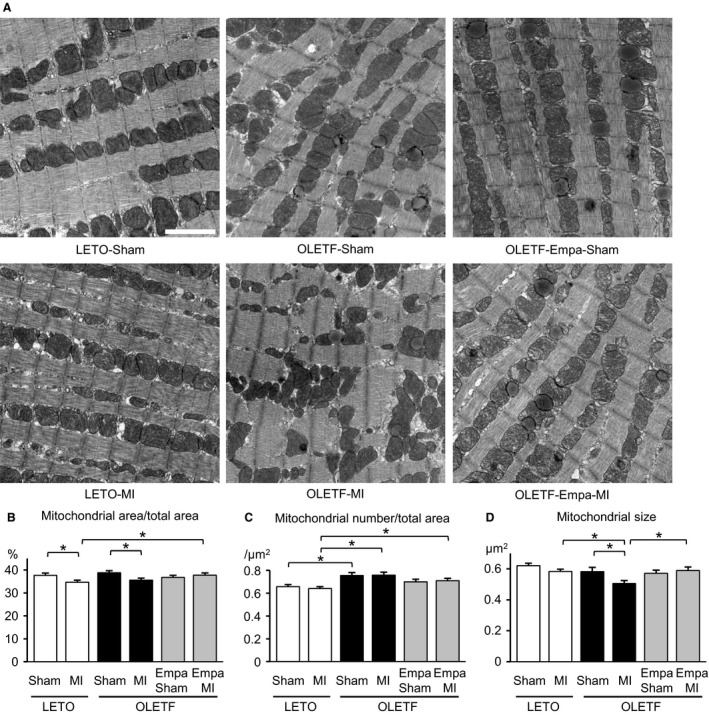
Effects of myocardial infarction and empagliflozin on mitochondrial number and size in the noninfarcted region of LETO and OLETF. (A) Representative electron micrographs from the noninfarcted myocardium of LETO, OLETF treated with a vehicle, and OLETF treated with empagliflozin (Empa) that were subjected to a sham operation or coronary artery ligation (MI). Scale bar = 2 *μ*m. Summary data of the percentage of total mitochondrial area (B), mitochondrial number per area (C), and the area of individual mitochondria (D) in 6 groups are shown. Ten images were taken from each heart, and data for 5–6 rats in each group were analyzed. *P < 0.05.

Myocardial tissues were fixed with 2.5% glutaraldehyde in 0.1 mol/L cacodylate buffer (pH 7.4) overnight at 4°C, followed by postfixation with 1% OsO_4_. After dehydration, the specimens were conventionally processed and examined under an electron microscope (H7650, Hitachi, Japan). For analyses of mitochondrial morphology and lipid droplets, 10 random fields from 2 blocks were captured at 15,000 × (9.3 *μ*m × 9.3 *μ*m) in each heart. A total of 50–60 images from 5 or 6 rats in each group were analyzed. For counting autophagic vacuoles including nascent and mature autophagosomes and autolysosomes, five randomly selected images in each heart were captured at 5000 × (28 *μ*m × 28 *μ*m) and data from five or six rats were compared. Data were expressed as number per 100 *μ*m^2^. Areas of individual mitochondria and lipid droplets were quantified by using ImageJ software (National Institutes of Health). In this study, we focused on interfibrillar mitochondria (Fig. 1A) because this population of mitochondria has been reported to be more susceptible than subsarcolemmal mitochondria to diabetes‐induced change in size (Dabkowski et al. 2009).

### mRNA quantification

Total RNA was isolated from frozen tissues by using an RNeasy Fibrous Tissue Mini Kit (Qiagen, Valencia, CA). First‐strand cDNA was synthesized using a SuperScript VILO™ cDNA Synthesis Kit (Life Technologies). DNA amplification was performed in ABI PRISM7500 (Life Technologies) by using Taqman Universal PCR Master Mix (Applied Biosystems, Inc). Taqman gene expression assays used in this study were as follows: Parkin (Rn00571787_m1), BCL2/adenovirus E1B‐interacting protein 3 (Bnip3) (Rn00821446_g1), and *β*‐actin (Rn00667869_m1). All assays were performed in duplicate and by the standard curve method using serial cDNA dilution. *β*‐Actin served as an internal control.

### Immunoblotting

Frozen tissue samples were homogenized in ice‐cold buffer (CelLytic™ MT Cell Lysis Reagent) containing protease and phosphatase inhibitor cocktails (Nacalai Tesque, Inc., Kyoto, Japan). The homogenate was centrifuged at 15,000 *g* for 15 min at 4°C to obtain the supernatant. Equal amounts of protein were analyzed by immunoblot assays using mouse monoclonal anti‐Mfn1 (Abcam, ab57602, 1:1000), rabbit polyclonal anti‐Mfn2 (Abcam, ab50838, 1:1000), mouse monoclonal anti‐OPA1 (BD Biosciences, 612606, 1:1000), rabbit polyclonal anti‐Fis1 (GeneTex, GTX111010, 1:1000), rabbit monoclonal anti‐phospho‐Ser616‐Drp1 (Cell Signaling Technology, #4494, 1:1000), rabbit monoclonal anti‐phospho‐Ser637‐Drp1 (Cell Signaling Technology, #6319, 1:1000), mouse monoclonal anti‐Drp1 (BD Biosciences, 611112, 1:1000), mouse monoclonal anti‐Bnip3 (Sigma Aldrich, B7931, 1:1000), and mouse monoclonal anti‐vinculin (Sigma Aldrich, V9131, 1:5000). Intensities of individual bands were quantified by using Image J software.

### Assay of tissue triglyceride level

Cardiac tissues were homogenized in 5% NP‐40/H_2_O solution. The samples were heated to 80°C in a heat block for 3 min and then cooled down to room temperature. This procedure was carried out one more time and samples in 1.5‐mL tubes were centrifuged for 2 min at 15,000 rpm. Supernatants were used for measurements of triglyceride (TG) by using a TG assay kit (Wako Pure Chemical, 290‐63701) in accordance with the manufacturer's instructions.

### Statistical analysis

Data are presented as means ± SEM. One‐way ANOVA and the Student–Newman–Keuls post hoc test were used to analyze differences in data among groups. The z‐test was used to determine the significance in proportions of mitochondria size between two groups (Picard et al. 2015). For all tests, *P* < 0.05 was considered statistically significant. All analyses were performed with SigmaStat (Systat, San Jose, CA).

## Results

### Empagliflozin decreased blood levels of glucose, total cholesterol, and triglyceride in OLETF

Under baseline conditions, OLETF showed greater body weight and higher blood glucose than those in LETO (Table 1). Empagliflozin did not change body weight but significantly decreased blood glucose level in OLETF. Under the conscious condition, heart rates were similar in LETO and OLETF and blood pressure tended to be higher in OLETF. Blood pressure in OLETF assigned to MI was slightly higher than those in other groups of OLETF, possibly due to inclusion of rats with large body weights by chance. There was no significant effect of empagliflozin on heart rate or blood pressure (Table 1).

**Table 1:** Metabolic and hemodynamic parameters before surgery after 12 h of fasting

	Body weight (g)	Blood glucose (mg/dL)	Heart rate (bpm)	mBP (mmHg)
LETO‐Vehicle				
Sham	536 ± 9	117 ± 3	325 ± 7	110 ± 5
MI	518 ± 9	124 ± 4	334 ± 8	112 ± 4
OLETF‐Vehicle				
Sham	629 ± 16^a^	179 ± 16^a^	320 ± 10	120 ± 3
MI	656 ± 8^a^^,^ c	152 ± 5^a^	322 ± 10	126 ± 5^a^
OLETF‐Empagliflozin				
Sham	639 ± 11^a^	114 ± 10^b^	325 ± 5	112 ± 1
MI	642 ± 9^a^^,^ c	121 ± 10	331 ± 7	118 ± 4

Values are means ± SEM. *N* = 10. bpm, beats per minute; mBP, mean blood pressure; MI, myocardial infraction.

a *P* < 0.05 versus LETO‐Vehicle sham.

b *P* < 0.05 versus OLETF‐Vehicle sham.

c *P* < 0.05 versus LETO‐Vehicle MI.

Ten rats were assigned to sham surgery in each of the LETO, OLETF, and empagliflozin‐treated OLETF groups, and none of sham‐operated rats died within 12 h after surgery. MI was induced in 11 LETO, 14 OLETF, and 12 empagliflozin‐treated OLETF, and the mortality rates during a period of 12 h after coronary occlusion were not significantly different between the three groups (9%, 28%, and 17%, respectively). At 12 h after MI, blood glucose levels were lower in empagliflozin‐treated OLETF (Table 2). Empagliflozin significantly decreased total cholesterol level in sham‐operated OLETF (Table 2). Serum TG levels were significantly higher in OLETF than in LETO as we previously reported (Miki et al. 2009; Hotta et al. 2010), and empagliflozin significantly reduced TG level in OLETF after MI. Heart rate under isoflurane anesthesia and respiratory support was lower in OLETF than in LETO as we previously reported (Kouzu et al. 2015; Murase et al. 2015) and was not affected by empagliflozin. Blood pressures were comparable in the study groups regardless of MI or empagliflozin treatment.

**Table 2:** Metabolic and hemodynamic parameters 12 h after surgery

	Blood glucose (mg/dL)	Total cholesterol (mg/dL)	Triglyceride (mg/dL)	Heart rate (bpm)	mBP (mmHg)
LETO‐Vehicle					
Sham	133 ± 5	95 ± 3	30 ± 7	356 ± 5	75 ± 3
MI	136 ± 8	105 ± 4	28 ± 6	350 ± 6	78 ± 2
OLETF‐Vehicle					
Sham	195 ± 16^a^	121 ± 11	176 ± 36^a^	253 ± 10^a^	83 ± 3
MI	222 ± 14^a^^,^ c	115 ± 7	225 ± 24^a^^,^ c	243 ± 9^a^^,^ c	76 ± 6
OLETF‐Empagliflozin					
Sham	99 ± 9^b^	78 ± 5^b^	120 ± 13	241 ± 9^a^, [Link]	79 ± 3
MI	116 ± 8^d^	98 ± 5	138 ± 16^d^	251 ± 6^a^^,^ c	84 ± 3

Values are means ± SEM. *N* = 6–10. bpm, beats per minute; mBP, mean blood pressure; MI, myocardial infraction.

a *P* < 0.05 versus LETO‐Vehicle Sham.

b *P* < 0.05 versus OLETF‐Vehicle Sham.

c *P* < 0.05 versus LETO‐Vehicle MI.

d *P* < 0.05 versus OLETF‐Vehicle MI.

### The number of smaller mitochondria was increased in OLETF

**Figure 2:**
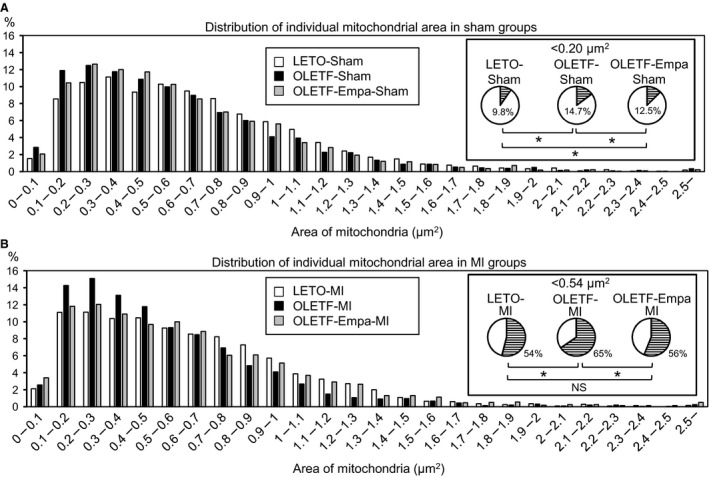
Frequency distribution analysis of individual mitochondrial areas: LETO versus OLETF. (A) Frequency distributions of areas of individual mitochondria in rats subjected to a sham operation. Pie charts show the proportion of mitochondria less than 0.20 *μ*m^2^ in each group. *P < 0.05 by the *z*‐test. (B) Frequency distributions of areas of individual mitochondria in rats subjected to coronary artery ligation (MI). Pie charts show proportion of mitochondria less than 0.54 *μ*m^2^ in each group. In each group, 2364 (LETO Sham), 2505 (OLETF Sham), 2466 (OLETF‐Empa‐Sham), 2546 (LETO‐MI), 2269 (OLETF‐MI), and 2201 (OLETF‐Empa‐MI) mitochondria from 50 to 60 images were analyzed. *P < 0.05 by the *z*‐test. NS = not significant.

In observation of the myocardium of LETO and OLETF by electron microscopy, significant changes in myofibrils, such as derangement or lysis of myofilaments, within cardiomyocytes were not detected in the hearts of OLETF or LETO with and without MI. Total mitochondrial areas were comparable in the sham‐operated groups (Fig. 1B). However, as shown in Figure 1C, the number of mitochondria per area was significantly increased in sham‐operated OLETF (OLETF‐Sham) compared with that in sham‐operated LETO (LETO‐Sham). The average of individual mitochondrial sizes tended to be smaller in the OLETF‐Sham group than in the LETO‐Sham group, but the difference did not reach statistical significance (Fig. 1D). Thus, frequency distribution analysis was performed for mitochondrial area data. As shown in Figure 2A, the frequency of a mitochondrial area of <0.20 *μ*m^2^ was significantly increased in OLETF‐Sham (14.7%) compared with that in LETO‐Sham (9.8%).

### MI‐induced reduction in the number of mitochondria in the remote noninfarcted region of the heart

**Figure 3:**
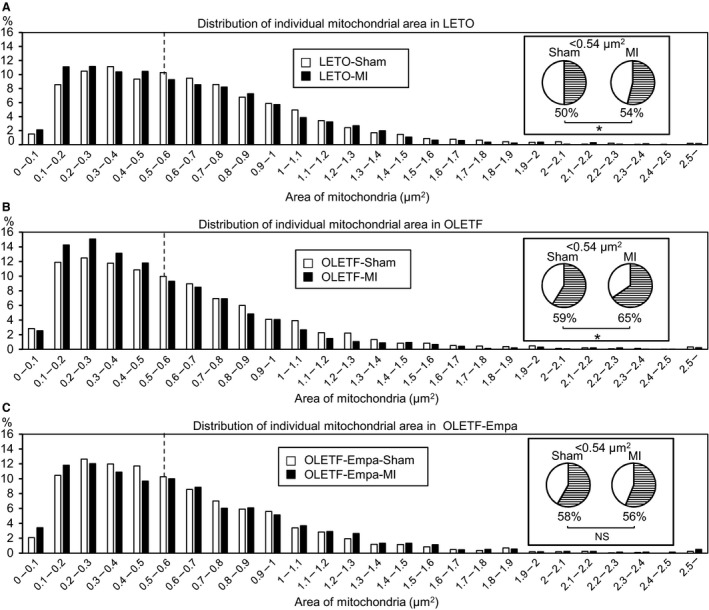
Frequency distribution analysis of impacts of myocardial infarction on mitochondrial size in the noninfarcted myocardium in LETO and OLETF. Comparisons of frequency distribution of individual mitochondrial area between the sham and myocardial infarction (MI) groups in LETO (A), OLETF treated with a vehicle (B), and empagliflozin (Empa)‐treated OLETF (C). Vertical dashed lines indicate a median value (0.54 *μ*m^2^) of mitochondrial size in the LETO sham group, and pie charts show the proportion of mitochondria less than 0.54 *μ*m^2^ in each group. As in Figure 2, 2364 (LETO Sham), 2505 (OLETF Sham), 2466 (OLETF‐Empa‐Sham), 2.546 (LETO‐MI), 2269 (OLETF‐MI), and 2201 (OLETF‐Empa‐MI) mitochondria from 50 to 60 images were analyzed in each group. *P < 0.05 by the *z*‐test. NS, not significant.

The total mitochondrial area in the noninfarcted region after MI of LETO hearts (LETO‐MI) was significantly smaller than that in LETO‐Sham, and such a reduction in the mitochondrial area after MI was observed in the noninfarct region of OLETF hearts (OLETF‐MI) compared with that in the control, OLETF‐Sham (Fig. 1B). The number of mitochondria was not significantly changed after MI in either LETO or OLETF (Fig. 1C). The average size of mitochondria was significantly smaller in OLETF‐MI than in OLETF‐Sham, while the difference between LETO‐MI and LETO‐Sham was not statistically significant (Fig. 1D). However, frequency distribution analysis indicated that the proportion of mitochondrial areas of <0.54 *μ*m^2^ (median of the area in LETO) was increased in the noninfarcted remote regions in both LETO and OLETF after MI (Fig. 3A and B). Furthermore, there was a significant difference in the average of mitochondrial size in the noninfarcted myocardium between LETO‐MI and OLETF‐MI (Fig. 1D), which was supported by the increase in frequency of a mitochondrial area of <0.54 *μ*m^2^ after MI in OLETF‐MI compared with that in LETO‐MI (Fig. 2B).

### Empagliflozin attenuated the MI‐induced reduction in mitochondrial size in OLETF

There was no significant difference between LETO‐Sham and empagliflozin‐treated/sham‐operated OLETF (OLETF‐Empa‐Sham) in total mitochondrial area, number of mitochondria, and average size of mitochondria (Fig. 1B–D). In contrast to the vehicle‐treated OLETF groups (i.e., OLETF‐Sham vs. OLETF‐MI), total mitochondrial area, number of mitochondria, and average size of mitochondria did not differ between the control group (OLETF‐Empa‐Sham) and the noninfarcted region after MI of the empagliflozin‐treated OLETF group (OLETF‐Empa‐MI). A lack of change in mitochondrial size after MI in empagliflozin‐treated OLETF was confirmed by the results of frequency distribution analysis (Fig. 3C).

### The number of autophagic vacuoles was maintained by empagliflozin after MI in OLETF

**Figure 4:**
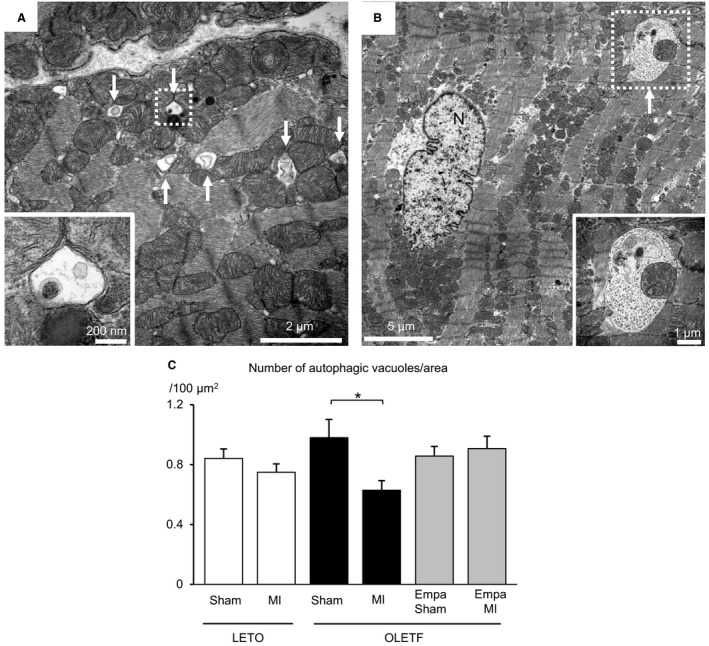
Effect of empagliflozin on autophagic vacuoles in noninfarcted myocardium. (A and B) Representative electron micrographs of autophagic vacuoles in the myocardium observed in LETO after the sham operation (A) and OLETF treated with a vehicle after the sham operation (B). Arrows indicate autophagic vacuoles. *N* = nucleus. (C) Average of the number of autophagic vacuoles per area in the myocardium from six groups of rats. Five images were taken from each heart, and data for five or six rats in each group were analyzed. *P < 0.05.

By the use of marker proteins of autophagy, we previously showed that activation of adaptive autophagy in the noninfarcted region of the heart 12 h after MI was significantly attenuated in OLETF compared with that in LETO (Murase et al. 2015). To confirm that previous finding by morphological data, we quantified the number of autophagic vacuoles in the noninfarcted myocardium after MI and its control myocardium by electron microscopy. As shown in Figure 4, autophagic vacuoles with diameters of approximately 0.5 *μ*m were observed in cardiomyocytes (Fig. 4A), being consistent with a previous report (Mizushima et al. 2002), and some of the vacuoles included mitochondria (Fig. 4B). There was no significant difference in the numbers of autophagic vacuoles per area among the three sham‐operated groups (Fig. 4C). The number of autophagic vacuoles was significantly smaller in the noninfarcted region of the heart than in the sham‐operated control in OLETF (OLETF‐MI vs. OLETF‐Sham). However, such an MI‐induced reduction in autophagic vacuoles was not observed in LETO and empagliflozin‐treated OLETF.

### Empagliflozin normalized Fis1 level and suppressed MI‐induced phosphorylation of Ser616‐Drp1 in OLETF

**Figure 5:**
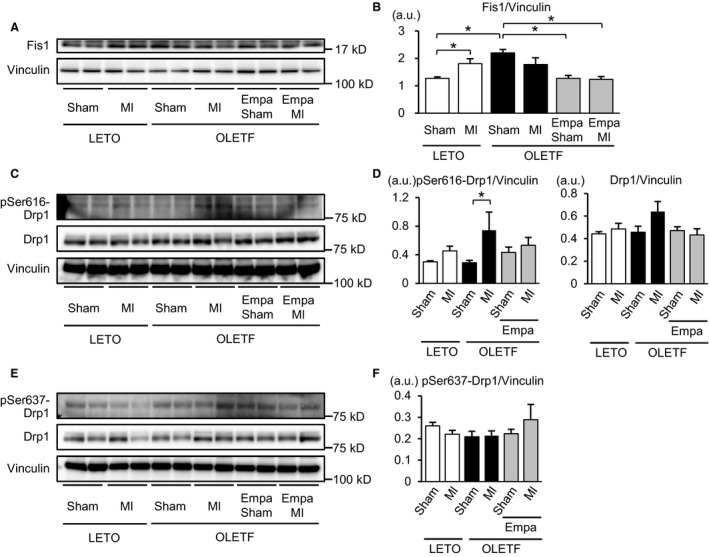
Effects of myocardial infarction on regulatory factors of mitochondrial fission. (A) Representative immunoblots for Fis1 in the noninfarct remote myocardium from LETO, OLETF treated with a vehicle, and OLETF treated with empagliflozin (Empa) that were subjected to a sham operation or coronary artery ligation (MI). (B) Summary data of Fis1 protein level normalized to vinculin. (C) Representative immunoblots for phospho‐Ser616‐Drp1 and total Drp1 in the noninfarcted myocardium from rats of six groups. (D) Summary data of levels of phospho‐Ser616‐Drp1 and total Drp1. (E) Representative immunoblots for phospho‐Ser637‐Drp1 and total Drp1. (F) Summary data of phospho‐Ser637‐Drp1 and total Drp1 normalized to vinculin level. *N* = 8 in each group. *P < 0.05. a.u., arbitrary unit.

**Figure 6:**
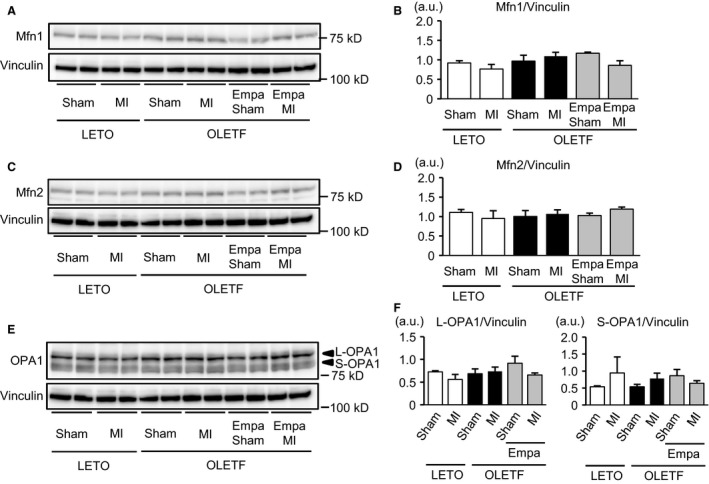
Effects of myocardial infarction on regulatory factors of mitochondrial fusion. (A) Representative immunoblots for mitofusin 1 (Mfn1) in the noninfarcted myocardium in LETO and in OLETF treated with either a vehicle or empagliflozin (Empa) that were subjected to a sham operation or coronary ligation (MI). (B) Summary data of Mfn1 protein levels normalized to vinculin. (C) Representative immunoblots for Mfn2. (D) Summary data of Mfn2 protein levels. (E) Representative immunoblot for OPA1. L‐OPA1: longer form of OPA1. S‐OPA1: shorter and soluble form of OPA1. The blot of vinculin is identical to that in (C) because blots of both Mfn2 and OPA1 were from the same membrane. (F) Summary data for quantification of L‐OPA1 and S‐OPA1 normalized to vinculin. *N* = 8 in each group.

To investigate how mitochondrial size is regulated in OLETF, we examined changes in machineries that regulate mitochondrial fission and fusion. The protein level of Fis1, which mediates mitochondrial fission (Liesa et al. 2009; Shenouda et al. 2011; Loson et al. 2013), was signif icantly increased in OLETF‐Sham compared with that in LETO‐Sham (Fig. 5A and B). Empagliflozin treatment canceled the increase in Fis1 protein level in sham‐operated OLETF. In LETO, MI significantly increased Fis1 protein level (Fig. 5A and B). However, such an MI‐induced upregulation of Fis1 was not observed in vehicle‐treated OLETF. Empagliflozin reduced Fis1 level in OLETF to the level in LETO and abolished Fis1 upregulation after MI (Fig. 5A and B). Levels of Ser616 phosphorylation in Drp1 were similar among the three sham‐operated groups (Fig. 5C and D). Phospho‐Ser616 level of Drp1 tended to be increased by MI in LETO, but the difference did not reach statistical significance. In contrast, Ser616‐Drp1 phosphorylation was significantly increased by MI in OLETF, the response of which was lost in empagliflozin‐treated OLETF. The slight increase in total Drp1 protein level by MI in OLETF did not reach statistical significance (Fig. 5C and D). There was no difference in levels of phospho‐Ser637‐Drp1 among the six treatment groups (Fig. 5E and F). There was no significant difference between levels of proteins that regulate mitochondrial fusion, Mfn1, Mfn2, and the long form of OPA1 (L‐OPA1) (Liesa et al. 2009), or between the levels of the cleaved shorter form of OPA1 (S‐OPA1), which is implicated in mitochondrial fission (MacVicar and Langer 2016), in the treatment groups (Fig. 6). The results suggest that upregulated mechanisms of mitochondrial fission in OLETF were suppressed by empagliflozin treatment.

### Bnip3 was upregulated by empagliflozin in OLETF

**Figure 7:**
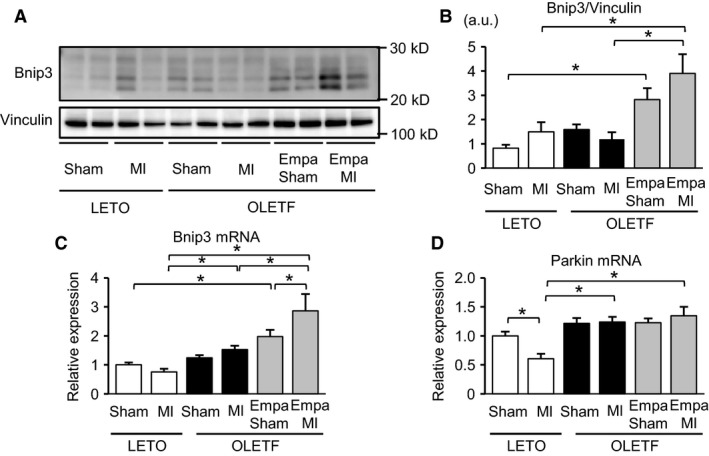
Alterations in Bnip3 and Parkin after myocardial infarction. (A) Representative immunoblots for Bnip3 in the noninfarcted myocardium in LETO and in OLETF treated with either a vehicle or empagliflozin (Empa) that were subjected to a sham operation or coronary ligation (MI). (B) Summary data of Bnip3 protein levels normalized to vinculin. *N* = 8 in each group. (C) Quantification of mRNA levels of Bnip3 in the myocardium. *N* = 8 in each group. (D) Parkin mRNA levels determined by a quantitative RT‐PCR method. *P < 0.05. a.u, arbitrary unit.

Empagliflozin decreased the mitochondrial number per area and the percentage of smaller mitochondria in OLETF (Figs. 1C and 2), suggesting that empagliflozin may promote elimination of small fragmented mitochondria via mitophagy. Thus, we assessed the effects of empagliflozin on levels of Bnip3 and Parkin expression. Bnip3 binds to LC3 as a mitochondrial receptor for autophagosomes to eliminate mitochondria (Zhu et al. 2013). Although the predicted molecular weight of Bnip3 is 21.5 kDa, multiple anti‐Bnip3 reactive bands were detected by immunoblotting due to phosphorylation‐dependent delay of electrophoretic mobility (Mellor et al. 2010). Bnip3 protein levels were comparable in LETO and vehicle‐treated OLETF regardless of MI (Fig. 7A and B). Empagliflozin treatment significantly increased Bnip3 protein levels in the sham‐operated control and noninfarcted myocardium after MI (Fig. 7A and B). Bnip3 mRNA levels were also upregulated by empagliflozin treatment in OLETF (Fig. 7C), suggesting transcriptional regulation by empagliflozin. The mRNA level of parkin, an E3 ubiquitin ligase that plays an important role in mitophagy (Nguyen et al. 2016), was significantly downregulated in the noninfarcted myocardium of LETO after MI but was unchanged in OLETF regardless of treatment with empagliflozin (Fig. 7D). The results support the notion that Bnip3‐mediated mitophagy was involved in the reduction of small mitochondria by empagliflozin in OLETF.

### Empagliflozin increased lipid droplets and triglyceride level in OLETF

**Figure 8:**
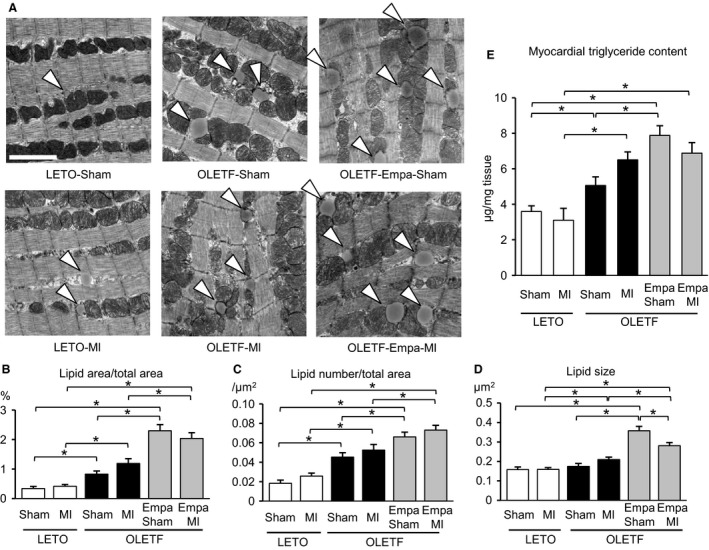
Lipid droplets in cardiomyocytes and tissue triglyceride levels: LETO versus OLETF. (A) Representative electron micrographs for lipid droplets (arrowheads) in the myocardium from LETO, OLETF treated with a vehicle, and OLETF treated with empagliflozin (Empa) that were subjected to a sham operation or coronary artery ligation (MI). Scale bar = 2 *μ*m. Data for the percentage of total area of lipid droplets (B), number of lipid droplets per area (C), and size of lipid droplets (D) in six groups are shown. Ten images were taken from each heart, and data for five or six rats in each group were analyzed. (E) Myocardial triglyceride levels (*μ*g/mg cardiac tissue) in the noninfarct myocardium. *N* = 9 in each group. *P < 0.05.

The total area and number of lipid droplets in cardiomyocytes were significantly increased in OLETF compared with those in LETO, and neither the area nor the number was different between the groups with and without MI (Fig. 8A–C). Empagliflozin treatment further increased the total area and number of lipid droplets in the control and the noninfarcted region after MI in OLETF (Fig. 8A–C). The individual size of lipid droplets was also increased by empagliflozin in OLETF (Fig. 8A and D). Levels of myocardial TG in treatment groups were almost in parallel to levels of total lipid areas determined by electron microscopy (Fig. 8E).

## Discussion

Dysfunction of mitochondria plays a major role in the pathogenesis of diabetes‐induced heart failure via increased reactive oxygen species (ROS), Ca^2+^ dysregulation, and impaired energy metabolism (Bugger and Abel 2010). In association of such functional abnormalities, various changes in mitochondrial morphology by diabetes have been reported. Although the relationship between functional abnormality and morphological changes of mitochondria in the natural history of diabetes mellitus has not been clarified, an increase in the number of mitochondria with a slight reduction in their size appears to precede more severe changes such as swelling of mitochondria, bizarre shape of mitochondria and mitochondria with disturbed cristae (Hayashi et al. 2003; Boudina et al. 2007; Bugger et al. 2008; MacDonald et al. 2011; Guglielmino et al. 2012; Montaigne et al. 2014). In the present study, the number of mitochondria was increased in OLETF compared with that in LETO (Fig. 1C), and analysis of the frequency distribution in individual mitochondrial areas revealed that small mitochondria were more prevalent in OLETF than in LETO (Fig. 2A). Although Montaigne et al. (2014) reported that mitochondrial fragmentation in the human diabetic myocardium was accompanied by a significant reduction in Mfn1 protein level, levels of Mfn1 and Mfn2 were unchanged in OLETF compared with those in LETO (Fig. 6A–D). In contrast, Fis1 level was increased in OLETF (Fig. 5A and B), being consistent with the results of a study showing that Fis1 expression in the endothelial cells was upregulated by diabetes, leading to increased mitochondrial fission (Shenouda et al. 2011). Interestingly, exposure of endothelial cells to a high concentration of glucose upregulated Fis1 expression and elevated mitochondrial levels of ROS (Shenouda et al. 2011). On the other hand, endogenous ROS and exogenous ROS have been shown to induce shortening and fragmentation of mitochondria in cardiac and noncardiac cells (Huang et al. 2016; Tsushima et al. 2017; Jezek et al. 2018). Generation of ROS in mitochondria in the diabetic heart is increased by multiple mechanisms (Bugger and Abel 2010), and OLETF at 25–30 weeks of age show mild diastolic dysfunction with preserved systolic function (Takada et al. 2012; Kouzu et al. 2015). Taken together, the results of the present study suggest that upregulation of Fis1, promoting mitochondrial fission, is one of the mechanisms of mitochondrial fragmentation in the diabetic heart, though multiple mechanisms are potentially involved in the morphological abnormalities of mitochondria induced by diabetes depending on the stage and etiology of the diabetes.

In contrast to mitochondrial changes during chronic ventricular remodeling several weeks after MI (Ide et al. 2001; Galan et al. 2016), their changes in the nonischemic myocardium exposed to acute hemodynamic load shortly after MI have not been characterized. Impaired mitochondrial respiration 4–6 weeks after MI was observed with or without mitochondrial fragmentation in the remote nonischemic region of the heart in small and large animal models of MI (Ide et al. 2001; Galan et al. 2016). The present study showed that mitochondrial size was modestly but significantly reduced in the nonischemic region as early as 12 h after MI in nondiabetic rats. This mitochondrial size change was accompanied by upregulation of Fis1 without a significant change in the phosphorylation of Drp1 or the expression of Mfn1, Mfn2, OPA1, or Bnip3. Interestingly, the acute change in mitochondrial size after MI was significantly enhanced in OLETF hearts. The level of Fis1 protein in OLETF was increased to the level in LETO after MI, and MI increased phosphorylation of Drp1‐Ser616, which promotes mitochondrial fission (Liesa et al. 2009). On the other hand, the number of autophagic vacuoles was significantly reduced after MI in OLETF (Fig. 4C). These findings suggest that Drp1 phosphorylation in addition to Fis1 upregulation mediates the augmented reduction in mitochondrial size after MI in diabetic hearts.

We do not have a clear explanation for how phospho‐Ser616 Drp1 level was increased after MI in OLETF. In the heart, Drp1 phosphorylation at Ser616 is increased by various stresses (Shirakabe et al. 2016; Xu et al. 2016; Tsushima et al. 2017; Xia et al. 2017), and this serine residue is phosphorylated by cyclin‐dependent kinase 1 (CDK1)/cyclin B (Taguchi et al. 2007), protein kinase C delta (Qi et al. 2011), extracellular regulated kinase (ERK) (Prieto et al. 2016), and Ca^2+^/calmodulin‐dependent kinase II (CaMKII) (Xu et al. 2016). CaMKII is activated in the noninfarct area after MI (Singh et al. 2012), and the level of oxidized CaMKII, an activated form of CaMKII, has been reported to be increased in the diabetic myocardium (Luo et al. 2013). Therefore, CaMKII may be a kinase that is responsible for Drp1 phosphorylation at Ser616 after MI in OLETF.

Our study conducted in parallel with the present study showed that treatment with empagliflozin improved the survival rate of OLETF during a period of 48 h after MI from 40% to 70%, and the protection was associated with increased ketone utilization in energy metabolism, preserved ATP level and restoration of SOD2 and catalase levels in the myocardium at 12 h after MI (Oshima et al. 2017). In the present study, empagliflozin prevented changes in both the number and size of mitochondria in OLETF (Fig. 1C and D) and also MI‐induced reduction in mitochondrial size (Fig. 1D and 2B). The MI‐induced reduction of autophagic vacuoles in the noninfarcted myocardium of OLETF was also prevented by empagliflozin (Fig. 4C). Although change in mitochondrial size is not necessarily causally related to tolerance of the cell to injury (Ide et al. 2001; Galan et al. 2016), the change in mitochondrial morphology from diabetic type to nondiabetic type by empagliflozin (Fig. 1) supports the notion that mitochondria are targets of cardiac protection afforded by empagliflozin.

Restoration of the number and size of cardiac mitochondria in OLETF by empagliflozin could be interpreted by two possible mechanisms. First, it is plausible that empagliflozin suppressed diabetes‐induced mitochondrial fission. Upregulation of Fis1 before and after MI (Fig. 5A and B) and MI‐induced phosphorylation of Drp1 at Ser616 in OLETF (Fig. 5C and D) were significantly suppressed by empagliflozin treatment, though proteins regulating mitochondrial fusion were unchanged (Fig. 6). Since Fis1 expression has been reported to be upregulated by hyperglycemia (Shenouda et al. 2011), reduction in plasma glucose level might have been involved in the effect of empagliflozin on Fis1 level. Suppression of the production of ROS also might contribute to the prevention of mitochondrial fission by empagliflozin in OLETF, since empagliflozin was found to reduce ROS level in the myocardium via upregulated SOD2 and catalase (Oshima et al. 2017). Second, it is possible that restoration of mitophagy contributed to the reduction of small mitochondria by empagliflozin in OLETF. This possibility is supported by the findings that empagliflozin increased Bnip3 expression (Fig. 7) and prevented MI‐induced reduction of autophagosomes in OLETF (Fig. 4C). Although our previous studies showed that impaired activation of an AMP‐activated protein kinase (AMPK) and hyperactivation of mTORC1 are involved in suppressed autophagy in OLETF (Murase et al. 2015; Muratsubaki et al. 2017), we could not detect significant effects of empagliflozin on AMPK or mTORC1 in OLETF in the noninfarcted myocardium (Oshima et al., unpubl. observation).

An SGLT2 inhibitor has been shown to attenuate nonalcoholic fatty liver disease in patients with type 2 DM (Ito et al. 2017) and to reduce liver TG level in diabetic mice (Suzuki et al. 2014). In contrast, we found that empagliflozin increased lipid droplets and TG level in the myocardium (Fig. 8). Administration of an SGLT2 inhibitor has been reported to increase the plasma level of free fatty acid (FFA) in patients with type 2 diabetes (Ferrannini et al. 2014) and in a high fat‐fed rat model (Yokono et al. 2014) due to enhanced lipolysis. Since the rate of fatty acid uptake into cardiomyocytes is dependent on the blood concentration of FFA, it is possible that the lipid accumulation induced by empagliflozin resulted from elevation of the circulating FFA level, possibly caused by the 24‐h fasting in the setting of our experiments. We speculate that reduced transport of FFA to the mitochondria might also be involved in the accumulation of lipid droplets in cardiomyocytes, because CPT1b expression was decreased by empagliflozin (Oshima et al., unpubl. data).

Whether promotion of lipid accumulation by empagliflozin is protective or detrimental for the diabetic myocardium is currently unclear. A lipid overload induced by overexpression of long chain acyl‐CoA synthetase 1 in the heart increased the generation of ROS and mitochondrial fission and reduced ventricular contractility (Tsushima et al. 2017). In contrast, lipid droplet accumulation induced by empagliflozin was associated with a reduced number of fragmented mitochondria (Fig. 1), no change in basal cardiac function evaluated by echocardiography, and improved survival rate after MI (Oshima et al., unpubl. observation). Recent studies have indicated that diacylglycerol and ceramide, but not TG, are responsible for cardiac lipotoxicity (Schulze et al. 2016). In addition, several studies have shown protective roles of lipid droplets in cells by storing TGs incorporated from cytotoxic saturated fatty acid (Listenberger et al. 2003), by buffering cytosolic Ca^2+^ overload during ischemia‐reperfusion (Barba et al. 2009) and by preventing the production of ROS via sequestration of fatty acids from excess oxidation (Kuramoto et al. 2012). Whether any of those protective aspects of lipid droplets plays a role in cardioprotection afforded by empagliflozin remains to be further investigated.

There are limitations of this study. First, since we did not directly determine mitochondrial functions, the relationship between changes in mitochondrial morphology caused by empagliflozin and changes in mitochondrial function remains unclear. As recently reviewed by Galloway and Yoon (Galloway and Yoon 2012) and by Dorn (Dorn 2016), there are multiple mutual interactions between mechanisms that regulate mitochondrial shape and mechanisms that regulate mitochondrial respiratory functions. Increased glucose and fatty acid uptake in cells potentially triggers the formation of a vicious cycle of mitochondrial dysfunction and dysregulation of mitochondrial morphology via their impacts on mitochondrial membrane potential and ROS production (Galloway and Yoon 2012). Thus, it is likely that normalization of mitochondrial morphology by empagliflozin in diabetic hearts reflects some improvement of mitochondrial function. Second, we examined the effect of empagliflozin on cardiomyocyte ultrastructure only at a very early phase after MI (i.e., 12 h after MI), and the long‐term effect of empagliflozin on mitochondria in the noninfarcted region after MI remains unclear. We selected 12 h after MI as a time point for tissue sampling since the survival rate during a period of 48 h after MI was markedly different between OLETF and LETO (i.e., approximately 30% vs. 80%) (Takada et al. 2012; Murase et al. 2015), resulting in significant selection bias in tissue sampling at ~48 h after MI. Nevertheless, the effect of empagliflozin on chronic ventricular remodeling after MI in diabetic hearts is an important issue and warrants further investigation.

In summary, treatment with empagliflozin normalized the size and number of mitochondria and increased lipid droplets in cardiomyocytes of a rat model of diabetes. It also prevented MI‐induced additional mitochondrial fragmentation that appeared to be mediated by both suppression of Fis1‐ and phospho‐Ser616‐Drp1‐regulated mitochondrial fission and restoration of impaired autophagy. Those effects of empagliflozin on mitochondrial morphology and number may be causally related to protection afforded by empagliflozin against heart failure in diabetic hearts.

## Conflict of Interest

This study is partly supported by a grant from Boehringer Ingelheim Pharmaceuticals.

## Data Accessibility
